# Threat learning impairs subsequent associative inference

**DOI:** 10.1038/s41598-022-21471-2

**Published:** 2022-11-07

**Authors:** Olivier T. de Vries, Raoul P. P. P. Grasman, Merel Kindt, Vanessa A. van Ast

**Affiliations:** 1 Department of Clinical Psychology, University of Amsterdam, Amsterdam, The Netherlands; 2 Department of Psychological Methods, University of Amsterdam, Amsterdam, The Netherlands; 3 Amsterdam Brain and Cognition, University of Amsterdam, Amsterdam, The Netherlands

**Keywords:** Physiology, Psychology

## Abstract

Despite it being widely acknowledged that the most important function of memory is to facilitate the prediction of significant events in a complex world, no studies to date have investigated how our ability to infer associations across distinct but overlapping experiences is affected by the inclusion of threat memories. To address this question, participants (n = 35) encoded neutral predictive associations (A → B). The following day these memories were reactivated by pairing B with a new aversive or neutral outcome (B → C_THREAT/NEUTRAL_) while pupil dilation was measured as an index of emotional arousal. Then, again 1 day later, the accuracy of indirect associations (A → C?) was tested. Associative inferences involving a threat learning memory were impaired whereas the initial memories were retroactively strengthened, but these effects were not moderated by pupil dilation at encoding. These results imply that a healthy memory system may compartmentalize episodic information of threat, and so hinders its recall when cued only indirectly. Malfunctioning of this process may cause maladaptive linkage of negative events to distant and benign memories, and thereby contribute to the development of clinical intrusions and anxiety.

## Introduction

The last two decades have seen a dramatic shift in how episodic memories are understood, from rigid, passive records of the past to flexible, actively constructed representations in service of the future^1,2^. This change in conceptualization is underlined by the recent surge in studies investigating relational memory and the neurocognitive mechanisms that enable it^3^. The ability to recombine information across distinct but overlapping episodes, referred to as *associative inference*, is considered a key feature of relational memory^4^. Crucially, it functions to inform us in novel situations when habits and memories of single experiences are insufficient to generate the predictions needed to guide action and decision making^5^. From an evolutionary point of view, the most valuable memory traces to construct and maintain are those that help predict aversive experiences, such that these can be avoided in the future. However, despite the central role that negatively arousing memories are thought to play in adaptive future behavior^6^, investigations of associative inference have rarely researched the effects of emotion^7^, or accounted for its fundamentally prospective purpose. It is thus unknown how associative inference is affected when one of the recombined memories constitutes a threatening experience, and whether such an effect is moderated by noradrenergic arousal.

Contemporary neuroscientific studies demonstrate that non-emotional memories of distinct experiences that share a common element are integrated at the representational level^8,9^. Importantly, these findings imply that the representations that facilitate associative inference are established before they are actually required^10^. As associations between emotionally arousing events and their learned predictors are privileged in memory^11^, it can be hypothesized that integration of threatening events with pre-existing related memories is prioritized, resulting in enhanced associative inference for judgements that include a memory of threat (prioritization hypothesis). In line with this idea, specific retroactive emotional enhancements have previously been demonstrated: recognition memory was enhanced for neutral items that later, through new learning, acquired emotional significance as instances of a semantic category which predicts a mild shock^12^. As for associative memories, Zhu et al.^13^ recently revealed that when one element of an existing memory is paired to an emotional stimulus, associations within the original memory are strengthened. Similar evidence comes from a study using monetary reward as reinforcing stimulus, showing that a hippocampus-dependent mechanism allows positive value to spread to reactivated memories, thereby subconsciously biasing subsequent decision making^14^. Experiments using higher-order threat conditioning paradigms have similarly demonstrated that conditioned responses generalize to stimuli that are only indirectly predictive of threat^15–18^. However, note that these studies were not designed to test the effect of threat-predictive value on associative inference, which is a declarative memory ability, as the emotionally relevant stimuli used (shock or monetary reward) overlapped with many episodes instead of being unique. As a result, such previous study designs do not enable testing of indirect associations across episodes (associative inference) with threat stimuli.

In sharp contrast with this prioritization hypothesis, research into the effects of negative emotion on episodic associative memory paints a different picture. Memory for associations between items is typically *impaired* when a negative stimulus is involved^19^. This effect is thought to be driven primarily by noradrenergic arousal^20^. Studies by Bisby et al.^21–23^ have shown that the presence of a negative element during encoding decreases the accuracy and coherence of subsequent associative memory, despite recognition memory for the negative element itself being enhanced. If the formation of cross-memory associations *between* reactivated memories and novel experiences relies on the same processes that bind elements *within* memories, associative inference following threat learning should be impaired. Specifically, the integrated representations that support associative inference may be affected by emotion in the same way that simple associative memories are (impairment hypothesis).

Here we hypothesized that predictive threat learning can, once consolidated, affect associative inference. However, as the literature is unclear on the direction of this effect, this was left open. We further hypothesized that, regardless of the direction, the magnitude of this effect is amplified by noradrenergic arousal responses during threat learning. To test whether and how threat learning impacts associative inference, we developed the ‘Predictive Relational Emotional Memory (PRE-Memory) paradigm’. Unlike most previous studies of emotional associative memory, we spread out learning and testing over several days. The effects of emotion on episodic memory typically require time to emerge^24^, as alteration via synaptic consolidation is a protein-synthesis dependent process^25^. Further, the formation of cognitive-map like overlapping representations and extraction of regularities from them has always been thought of as a time-dependent process^26,27^. Another core difference with earlier work is that here, the neutral and emotional elements which form an episode were presented sequentially, rather than simultaneously. This ‘episodic threat learning’ procedure is likely to elicit defensive preparations to actively predict and cope with impending threat, triggering motivational systems for future-oriented action^28^. Specifically, participants first encoded neutral premise memories (A → B), which on the following day were linked to multimodal stimuli that were either highly arousing or neutral (B → C_THREAT/NEUTRAL_). Then, on the third and final day, participants made associative inferences, recombining the indirectly linked elements across premise memories (A → C_THREAT/NEUTRAL_). Noradrenergic arousal responses to B and C items during episodic threat learning were indexed by means of pupillometry^29^. Employing the novel PRE-Memory paradigm, the present study sheds light on the functioning of a complex feature of episodic memory in those situations when it may matter the most.

## Methods

### Participants

Due to the current unavailability of tools for determining the necessary sample size in logistic multilevel regressions, no a priori power analysis was conducted. Instead, we reasoned that the sample size should be (1) comparable to those reported in key studies that inspired this experiment, and (2) sufficient to reliably estimate parameters in a two-level model. Bisby et al. consistently demonstrated impairments in emotional associative memory across three experiments with sample sizes between 17 and 27^21^, whereas enhancements of neutral memories following threat learning have been shown in samples of 30 participants^12,13^. We thus sought to include at least 30 participants, such that both an impairing or enhancing effect of threat on associative inference could be detected. Moreover, sample sizes of 30 or more participants are likely to yield unbiased estimates in multilevel models^30^. Anticipating some drop-out and missing data for the pupil measure, 47 healthy individuals were recruited via the university’s online system and gave written informed consent to participate in this study.

Exclusion criteria as assessed via a screening based on self-report were recreational drug use at a frequency higher than once a month, average consumption of 21 or more units of alcohol per week, having experienced trauma, and having received treatment for a mental disorder listed in the DSM-5 by a psychologist or psychiatrist in the past year. Participants were rewarded either with course credits or 45 euros for completing the experiment, or according to the total amount of time spent in the lab in case of dropping out. Due to the high aversiveness of the picture-sound combinations on day two, it was emphasized that they were free to quit the experiment at any time without having to state a reason. Ten participants made use of this option on day two. Additionally, one participant aborted the computer task on day two by accidentally pressing the escape button, and another missed the appointment for the third day. The final sample thus includes data of 35 participants (mean age = 21.4, SD = 2.6, range = [19–28]; 24 women) prior to analysis. This study was performed in accordance with the Declaration of Helsinki and approved by the local ethics committee of the University of Amsterdam.

### Materials

#### Stimuli

We selected 80 neutral pictures of objects from the Bank of Standardized Stimuli (BOSS)^31^ to function as A and B stimuli. Forty C stimuli, 20 emotionally negative and 20 neutral, were partly chosen from the Nencki Affective Picture System (NAPS)^32^, and supplemented with copyright-free photos from the internet. Each C stimulus in the threat condition was matched to one in the neutral condition in terms of its content to control for memory effects of complexity and semantics. For example, an image of a fatal car crash in the threat condition was matched with an image of a car safely driving on the freeway in the neutral condition. For each C stimulus a corresponding sound was found from either the International Affective Digitized Sounds (IADS) database^33^, or The Freesound Project; a collaborative, open-source repository of audio content under a creative commons license (https://freesound.org/). These were selected to meaningfully match the image (e.g., image of badly broken leg—sound of snapping celery) to better emulate a real-life, multi-modal experience, thus enhancing the potential of the aversive stimuli to evoke ecologically valid physiological responses.

Two important adjustments were made to the stimuli. First, the mean luminance of all images was set equal using the color adaptation of the SHINE toolbox for MATLAB^34^. This diminishes the potential influence of low-level visual features of the stimuli that may confound pupil data^35^. Second, the maximum amplitude of each sound file was set such that it never exceeded 72 decibels in our lab setting, so pupil effects across conditions could not be explained as the result of loud noises^36^.

#### Experimental tasks and procedures

**Figure 1:**
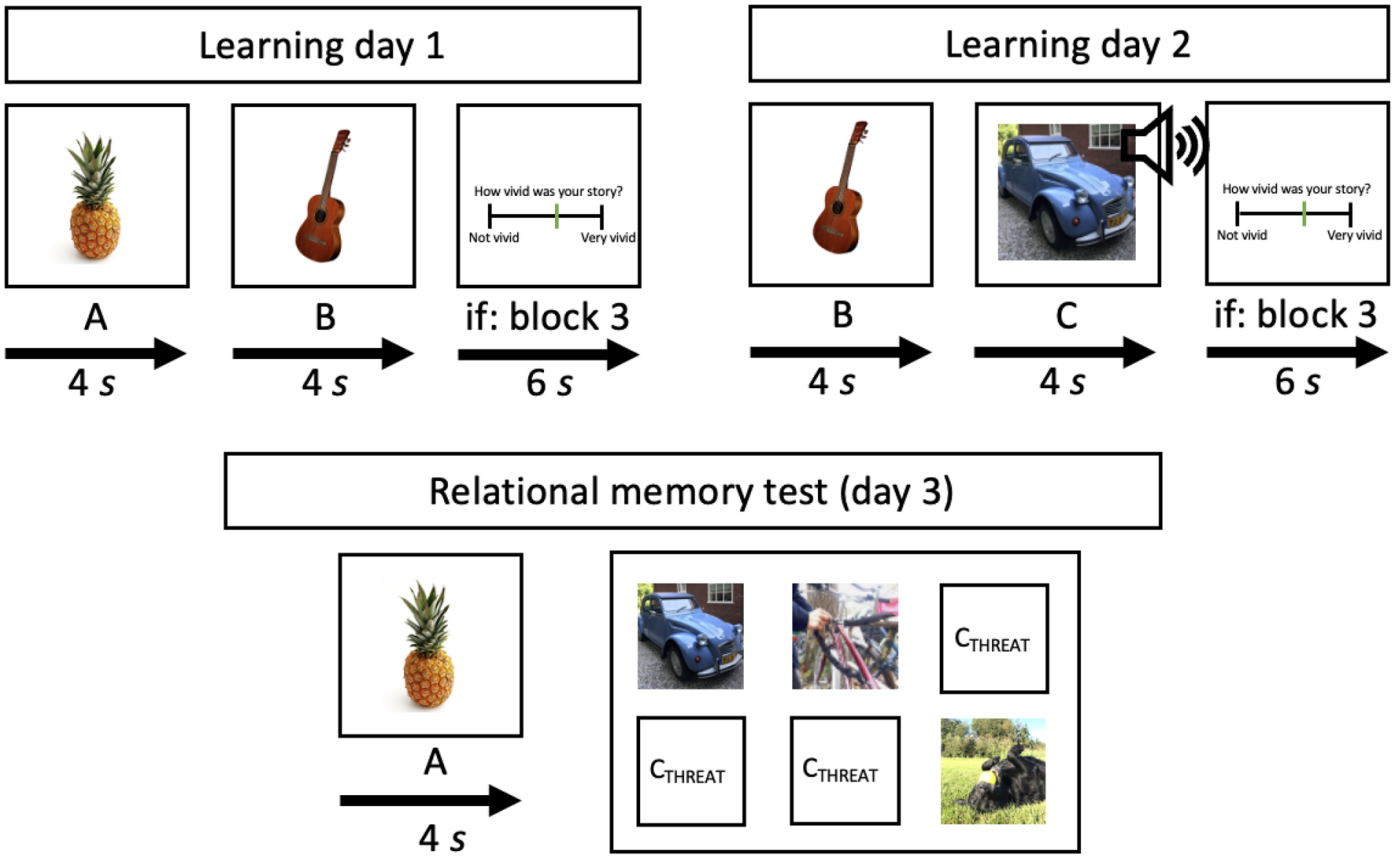
Overview of task structure on both learning days and the associative inference test phase on day 3. In this example the pineapple (A) is presented first, followed by the guitar (B). Then on day 2, each B item is presented and followed by a picture/sound combination (C) that can either be neutral or aversive. All pairs on both day 1 and 2 are presented a total of 3 times. During the last block of pair presentations on each day participants are asked to indicate the vividness of their imagined story for each pair. Finally, during the associative inference test, A items are presented followed by a six-alternative forced-choice test in which the correct C item must be selected using the numpad on the keyboard. The options belonging to the threat condition are shown schematically to protect the copyrights of the creators of the stimuli we used. Pictures representing stimulus A and B are examples from bank of standardized stimuli.

The different days of the PRE-Memory paradigm mirror the three distinct phases of a sensory preconditioning experiment^37^, and took place across three consecutive days (see Fig. 1). Note that despite this similarity in procedure for the first two phases of the experiment, the interest for the third phase here is declarative associative inference and how it is affected by threat, rather than conditioned physiological responses to the preconditioned stimulus. On the first day, neutral predictive associations are learned between pairs of stimuli (A → B). Then on the second day, of each pair, one (B) is used as a conditioned stimulus (CS) for associative learning, with each either neutral or aversive C item functioning as a unique, episodic, unconditioned stimulus (US; B → C). Finally, on the third day, we assessed participants’ ability to recombine information from both learning days by presenting each A item from day 1, which has become indirectly predictive of either a neutral or aversive C item on day 2 (A → C?). The experiment was programmed such that each participant was shown uniquely randomized combinations of A → B → C stimuli. Each session took place in the same lab room where the lighting was kept at the maximum level so baseline pupil diameters would be low, leaving room for dilation in response to aversive stimuli.

##### Learning (day 1)

On the first day participants were informed that the goal of the experiment is to study their ability to vividly imagine stories involving different picture combinations. This was to obscure the fact that the primary interest was memory, and so prevent the use of deliberate and variable learning strategies. After reading the information brochure and signing an informed consent form, participants took place in front of the computer screen. The experimenter read out the instructions for both the first associative learning task under the guise of an ‘imagination task’^38^. Specifically, participants were instructed to use each pair of presented stimuli to vividly imagine stories in which they themselves play a central role, either in a first person narrative or as an observer of an event. By having participants actively simulate events involving themselves and the pairs of stimuli, the memories containing each association are more likely to have the characteristics of “what, where, and when” that define episodic memory^39^. Participants were shown 40 pairs (A → B) of sequentially presented pictures. Each trial started with a fixation cross presented for 500 ms, after which stimulus A was shown for 4 s, immediately followed by stimulus B for another 4 s. Intertrial intervals (ITI) randomly varied between 8, 9, 10, 11, or 12 s, with an average of 10 s. Following presentation of all 40 unique pairs, they were all presented again in randomized order during a second and third learning block. Participants were given 1-min breaks between learning blocks. In the third block they were asked to indicate the vividness of each of their imagined stories on a visual analogue scale (VAS) ranging from 0 (‘not at all vivid’) to 100 (‘very vivid’). Next, participants completed an associative recognition test in which each A item was presented for 4 s, after which they were asked to select the associated B item from 6 options on the screen. Instructions for both tasks were repeated on the computer screen. At the end of the session a brief exit questionnaire was conducted inquiring after the participants’ motivation to comply with task instructions.

##### Threat learning (day 2)

The associative learning task of day two was almost identical in structure and instructions to that of the previous day, but very different in terms of the presented stimuli. First, the stimuli presented were now B → C pairs, meaning that each first image presented (B) was the second of a pair seen the day before. Moreover, the second item of each pair (C) could either be neutral or aversive (threat), and was accompanied by a corresponding sound played through headphones. This way, half of the 40 A → B pairs from the first day were ‘extended’ with an aversive C item (A → B → C_THREAT_), whereas the other half were newly associated with a neutral control C item (A → B → C_NEUTRAL_). Participants received the same instructions as the first day: to use each pair of presented stimuli to vividly imagine stories in which they themselves play a central role, but that now, some of these images could involve aversive images and sounds. Participants were not explicitly told that each first picture of a pair (B) would be the second picture of a pair they were presented yesterday, nor to actively reactivate its pre-existing associate (A). Participants were further asked to rest their head in a chinrest, and to minimize head movements during the experimental tasks so as to not interfere with pupil measurements. As on the first day, after presentation of all 40 B → C pairs, these were repeated in a second and third learning block. Additionally, in the first learning block participants were asked to indicate the valence (‘negative’ to ‘positive’) and arousal (‘calm’ to ‘tense’) induced by the stories they imagined with each pair of items on two separate VASs from 0 to 100. ITI duration and randomization were the same as the previous day, and participants were again given 1-min breaks between learning blocks. A maximum of three B → C pairs from the same condition were presented consecutively. After encoding, memory for all B → C pairs was tested in the same way as A → B pairs were on day one, and the session again ended with an exit questionnaire, which this time also included questions inquiring after the participants’ subjective emotional experience of the task.

##### Associative inference test (day 3)

On the final day participants first performed the associative inference test, followed by two associative recognition tests for the associations that had been learned on day one (A → B) and two (B → C) of the experiment. They received verbal and written instructions for each test separately. During the associative inference test, each A item was presented for 4 s followed by a self-paced, six-alternative forced-choice test in which participants had to select the C item it is indirectly associated with through a shared B item. Participants would use the numbers one to six on the numpad to indicate their answer for each trial. All of the lures presented during each test trial were other C stimuli that had been presented during the experiment and selected such that three were from the neutral condition and three from the threat condition. All C pictures were used equally often as lures. Following the associative inference test, the premise memories A → B and B → C were tested. The trial structure of these tests was identical to that of the associative inference test, except that the cue was presented for only 1 s. Finally, after the last exit questionnaire, again inquiring after their motivation to comply with the instructions of the tasks, participants were debriefed on the true objectives of the experiment.

### Data analysis

#### Acquisition and pre-processing

Behavioral data acquisition was performed using Presentation software (Neurobehavioral Systems Inc., Berkeley CA). Pupil data during day 2 was collected using a Tobii Pro Nano eye tracker set at a sampling rate of 60 Hz. The resulting time series were preprocessed using the Python programming language^40^ by (1) locating all samples registered by the eyetracker as missing values (NaN) as a result of participants blinking, looking away, or technical errors, and setting the samples 100 ms before and after to also be NaN, (2) linearly interpolating around these NaN values, and (3) applying a band-pass filter (0.01–6 Hz, third-order Butterworth).

Following these first steps, pupil responses were quantified for B and C stimuli by computing the mean value in the frame of interest: In case of the B stimuli, which here function as conditioned stimuli, this was the final second of presentation, as anticipatory fear-responses are most likely to be picked up just before the fear-invoking stimulus^41^. For C stimuli, we looked at the final 2 s of stimulus presentation, where the emotional response is at its peak^42^. For a trial value to be deemed of sufficient quality to be included in the analyses, both the mean value for a frame of interest and its corresponding baseline had to be computed on the basis of at least 50% non-NaN values, or otherwise it was set to missing. The mean values for each frame of interest were subtracted from the mean pupil width during a baseline of 500 ms before stimulus onset^29^. Participants for whom over 50% of trials in either condition were excluded based on this criterion were excluded from the pupil data analyses altogether.

#### Manipulation and premise checks

##### Premise associations

Following the associative inference test (A → C) on day 3 of the experiment, participants completed associative recognition tests for premise memories A → B and B → C, allowing for the specific selection of those A → C test trials for which the memories on which they are based have been retained^43^. Whether this resulted in an equal distribution of trials across conditions was assessed by means of an independent t-test. Furthermore, we assessed differences in associative memory between conditions immediately following learning on day one and two, also by means of independent t-tests.

##### Arousal responses to aversive stimuli and transfer to predictors

Two Condition (threat, neutral) × Block (one, two, three) repeated measures ANOVAs were carried out to test whether the aversive C stimuli were successful in evoking an arousal response, and whether these carried over to the B stimuli in the second and third block following learning. For the former, average pupil responses to the C stimuli were used as dependent variable, while for the latter, average pupil responses to B stimuli were used.

#### Primary analyses

We employed multilevel regression analyses, a modelling strategy that allows for hypothesis testing at the level of individual memories whilst taking into account the nested structure of memory trials within participants, for each of the main research questions posed here. As the dependent variable of each analysis is the binary outcome of the associative inference test trials which can either be correct or incorrect, we ran logistic multilevel regression models to test each hypothesis by means of the lme4 package for R^44^. The parameters estimated by logistic regression are changes in the natural logarithm of the predicted odds, which determines the likelihood of a discrete event happening. Odds can be converted to a proportion by calculating $\frac{Odds}{Odds+1}$. For example, if the predicted odds for correct associative inference are 4 this means the model predicts 4 correct inferences for every incorrect one, (or 80%), and the log odds ratio (log OR) would be ln(4) ≈ 1.39. All continuous predictor variables were subject-mean centered, meaning that results can be interpreted as effects of within-subject predictor variance.

Importantly, trials included in the analyses were only those for which both premise associations (A → B and B → C) were retained on the day of associative inference testing. This means that the results are to be interpreted as the effects of threat learning, arousal, and encoding vividness for instances in which the memories required to make an A → C judgement are readily available. In other words, any effects of threat on associative inference cannot be explained by differences across conditions in the retention of premise memories.

##### Effect of threat learning on associative inference

To test the hypothesis that threat learning affects future associative inference, we ran a multilevel logistic regression model with condition as the only predictor variable, where the log odds ratio of the predicted mean response accuracy (*ln(odds_hit)*) for trial *j*, nested in participant *i*, is:

$$\ln (odds\_hit_{ij} ) \, = \, \mu \, + \, \alpha_{i} + \, \beta_{1} \left( {Threat} \right)_{ij} .$$

The neutral condition was set as the reference category, meaning that the intercept *μ,* or grand mean, can be interpreted as the predicted average log odds ratio for making a correct associative inference for this condition. Individual variance in baseline performance, or likelihood of accurate associative inference in the neutral condition, is captured by the random intercepts, *α*_*i*_*.* The coefficient *β*_1_, then represents the difference in predicted average log odds ratio for threat trials relative to neutral trials. If the *β*_1_ parameter is significant, this indicates an effect of threat learning. A positive value for *β*_1_ would be evidence for an enhancing effect of threat, whereas a negative value would suggest an impairment.

We additionally tested whether reaction times (RT) differed between conditions, and whether this effect differed for correct and incorrect associative inferences, by means of a linear multilevel regression with two categorical predictors: condition and correctness, with neutral and incorrect as reference categories, respectively. The model was thus specified as follows:

$$RT \, = \mu \, + \, \alpha_{i} + \, \beta_{1} \left( {Threat} \right)_{ij \, + } \beta_{2} \left( {Correct} \right)_{ij \, + } \beta_{3} \left( {Threat \, \times \, Correct} \right)_{ij} .$$

Here, *β*_1_ is the estimated difference in reaction times for threat relative to neutral trials, and *β*_2_ between correct trials relative to incorrectly answered trials. *β*_*3*_ is the additive difference for trials that are both in the threat condition and have been answered correctly. Finally, we ran a repeated measures ANOVA of RTs with Condition (threat, neutral) and Associative test type (inference A → C, association A → B, association B → C) to assess whether associative inferences across memories were as fast as associative recognition judgements within memories^9^, and whether this differed between conditions.

##### Noradrenergic moderation of threat learning’s effect on associative inference

To assess whether arousal evoked by the aversive C stimuli (i.e., the US stimuli) was related to the possible effect of threat memories on associative inference (be it enhancement or impairment), pupil responses upon first encounter of C in the first block were then added as moderators of the effect of condition:

$$\ln (odds\_hit_{ij} ) \, = \, \mu \, + \, \alpha_{i} + \, \beta_{1} \left( {Threat} \right)_{ij} + \, \beta_{2} \left( {Arousal} \right)_{ij} + \, \beta_{3} \left( {Threat \, \times \, Arousal} \right)_{ij} .$$

Since arousal, operationalized as pupil dilation, is a continuous variable, *β*_2_ represents the corresponding slope for predicting the log odds ratio of accurate associative inference. The neutral condition is again used as reference, meaning that *β*_3_, the slope of the arousal × condition interaction, represents the difference in slopes for the effect of arousal in threat trials relative to neutral trials.

#### Secondary analyses

##### Controlling for vividness of premise memories

The extent by which threat learning affects associative inference may depend on the subjective encoding vividness of the original episodes that form the basis for a subsequent judgement. Emotionally arousing events are well-known to produce vivid memories^45^, which may affect associative memory strength. Thus, to control for effects of premise memory vividness, which may differ across conditions, participants gave a subjective vividness rating for premise memories on both learning days. We tested whether vividness moderates the effect of threat learning on subsequent associative inference by adding the vividness score for day 1 and day 2 to the model, specified as a three-way interaction with condition:

$$\begin{aligned} \ln (odds\_hit_{ij} ) = & \, \mu \, + \, \alpha_{i} + \, \beta_{1} \left( {Threat} \right)_{ij} + \, \beta_{2} \left( {Vividness\_D1} \right)_{ij} + \, \beta_{3} \left( {Vividness\_D2} \right)_{ij} \\ & + \beta_{4} \left( {Threat \, \times \, Vividness\_D1} \right)_{ij} + \, \beta_{5} \left( {Threat \, \times \, Vividness\_D2} \right)_{ij} \\ & + \beta_{6} \left( {Vividness\_D1 \, \times \, Vividness\_D2} \right)_{ij} \\ & + \beta_{7} \left( {Threat \, \times \, Vividness\_D1 \, \times \, Vividness\_D2} \right)_{ij} . \\ \end{aligned}$$

The vividness variables for both days, Vividness_D1 and Vividness_D2, are continuous variables, their respective main effects indicated by slopes *β*_2_ and *β*_3_. As is the case in the previous analyses, the interaction parameters *β*_4_ and *β*_5_ indicated the differences in the effect of premise memory vividness for threat trials relative to neutral trials for both encoding days. Finally, parameter *β*_6_ represents the interaction effect of premise memory vividness, whereas *β*_7_ indicates how that effect is different for threat trials compared to neutral.

##### Effects of threat learning on premise memories

Applying the same modelling strategy used to analyze the effect of threat learning and arousal on associative inference, we tested whether the original memories (A → B) were strengthened by the novel threat associations (B → C), which would be in line with earlier studies finding evidence of emotional tagging^12,13^. Similarly, we tested whether the associations between novel memory elements (B → C) differed between threat trials and neutral controls. Similar to our predictions on the effects of threat on associative inference, here we expected either an enhancement due to the predictive value for threatening C-items acquired by B-items, or an impairment in line with earlier studies towards the effect of emotion on associative learning^23^. Note that now, *Odds_hit*_*ij,,*_ refers to the binary outcome (correct or incorrect) of trials during the associative recognition test of associations from day one or day two, assessed on day three of the experiment.

$$\ln (odds\_hit_{ij} ) \, = \, \mu + \alpha_{i} + \beta_{{1}} \left( {Threat} \right)_{ij} .$$

## Results

### Manipulation checks

#### Premise memories

**Figure 2:**
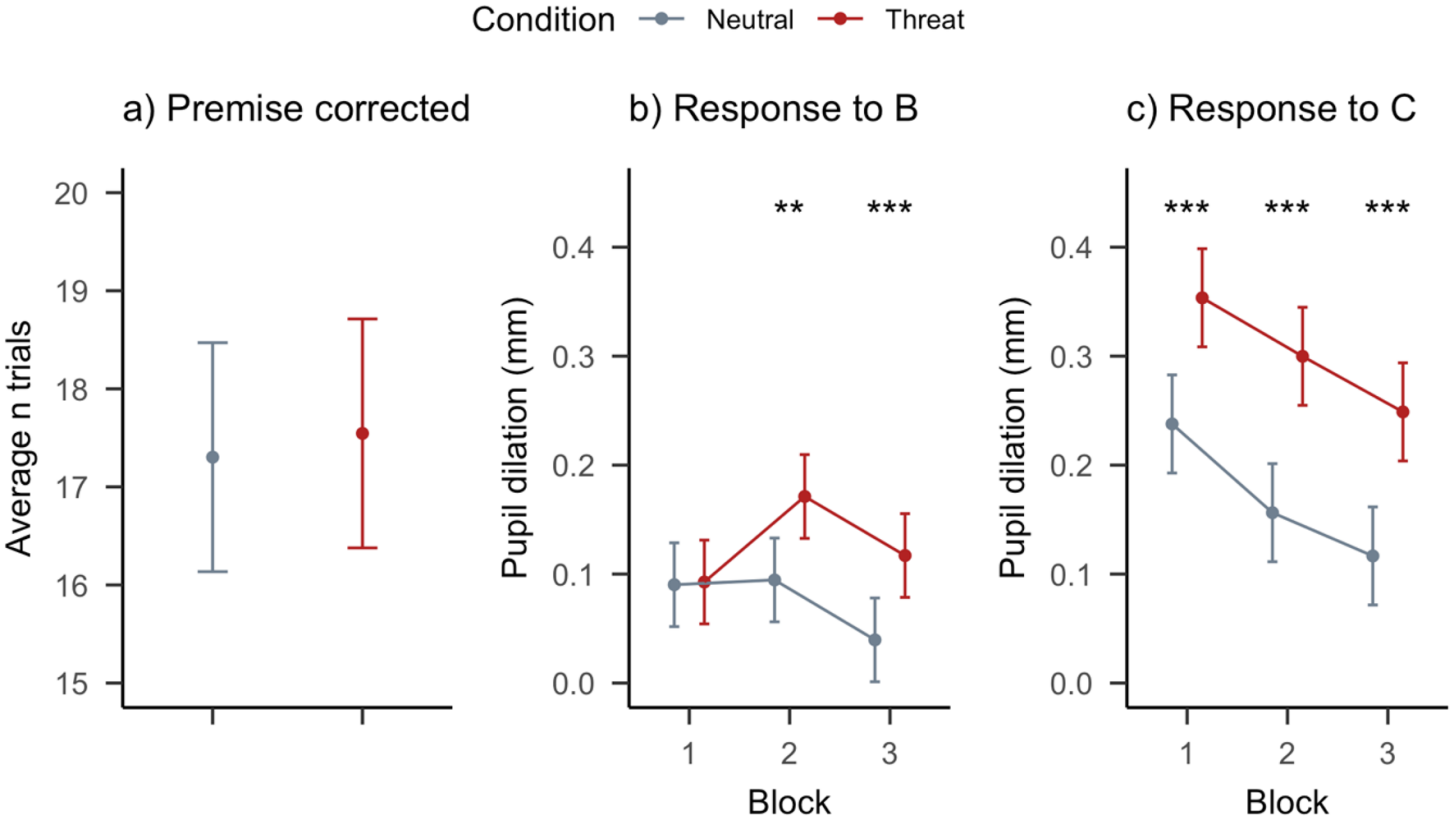
Analyses to validate the novel PRE-Memory paradigm. (**a**) In total, participants correctly remembered both premise memories for an average of 34.85 out of 40 associative inference trials. These were divided equally across conditions. (**b**) Pupil responses to neutral B stimuli that predicted an aversive or neutral C stimulus did not differ initially in block 1, but diverged after the predictive associations were learned over the course of blocks 2 and 3, indicating successful threat acquisition. (**c**) Pupil responses to the aversive C stimuli did not habituate—they remained consistently higher than the neutral ones throughout the experiment, even though pupil responsiveness gradually decreased over learning blocks. Error bars represent 95% confidence intervals. Asterisks indicate statistically significant differences between conditions (*p < 0.05, **p < 0.01, and ***p < 0.001).

Binomial tests for each individual in the sample revealed that 2 participants did not perform significantly above chance level (1/6), and were excluded from further analysis. Following selection of only those associative inference trials for which both premise memories were retained on day 3, an average of 34.85 trials (SD = 6.64) out of 40 remained for each participant (see Fig. 2a). There was no statistical difference in how these were distributed across the two conditions (*t*_32_ = 1.13, *p* = 0.407).

#### Pupil responses to C items and their predictors (B)

To assess whether the aversive stimuli were successful at inducing physiological arousal, we first ran a 3 × 2 repeated measures ANOVA using learning Block and Condition as within-subject factors to investigate individuals’ average pupil dilation to C stimuli (see Fig. 2c). This revealed a strong main effect of Condition (*η*_*p*_^2^ = 0.43, *CI*_90_ = [0.33, 0.52], *F*_1,120_ = 91.58, *p* ≤ 0.001), indicating that the aversive C stimuli indeed brought about a state of heightened arousal relative to the neutral stimuli. A main effect of Block (*η*_*p*_^2^ = 0.19, *CI*_90_ = [0.09, 0.29], *F*_2,120_ = 14.46, *p* < 0.001) further implies that overall, pupil responsiveness decreased over time as participants performed the learning task. The absence of a Condition × Block interaction (*η*_*p*_^2^ = 0.00, *CI*_90_ = [0.00, 0.00], *F*_2,120_ = 0.04, *p* = 0.958) indicates that emotional responses remained consistent across learning blocks and did not habituate towards the level of the neutral trials.

Then, to assess whether the emotional charge of ‘C’ items transferred to the neutral ‘B’ component of each trial, a repeated measures ANOVA with the same within-subject factors was conducted, now however to explain pupil dilation in response to B stimuli (see Fig. 2b). Critically, we found a statistical interaction between learning Block and Condition (*η*_*p*_^2^ = 0.07, *CI*_90_ = [0.01, 0.15], *F*_2,110_ = 4.14, *p* = 0.019), indicating that the effect of threat differed across learning blocks. Planned comparisons showed that pupil responses to ‘B’ stimuli that predict aversive ‘C’ stimuli were not higher relative to neutral controls in block 1 (difference = 0.00 mm, *CI*_95_ = [− 0.04, 0.04], *t*_110_ = 0.10, *p* = 0.924), but elicit greater pupil responses in the second block (difference = 0.07 mm, *CI*_95_ = [0.03, 0.11], *t*_110_ = 3.39, *p* = 0.001), and even more so in the third learning block (difference = 0.08 mm, *CI*_95_ = [0.04, 0.12], *t*_110_ = 3.81, *p* < 0.001). We again found a main effect of Condition (*η*^2^ = 0.14, *CI*_90_ = [0.05, 0.24], *F*_1,110_ = 17.73, *p* < 0.001) implying generally larger anticipatory pupil responses preceding aversive events, and a main effect of Block (*η*_*p*_^2^ = 0.13, *CI*_90_ = [0.04, 0.23], *F*_2,110_ = 8.03, *p* < 0.001).

These results confirm that the ‘C’ stimuli presented on the second day of the experiment were successful at evoking acute pupil dilation responses. Moreover, the increase in pupil responses to B items predictive of threat over learning blocks demonstrates that the episodic threat conditioning manipulation was successful. Together, these findings suggest that the present paradigm is suitable for investigating the respective effects of threat learning and acute noradrenergic arousal on associative inference.

#### Subjective emotion and vividness ratings

Consistent with our pupil dilation results, participants’ subjective self-reported affective responses to their own imagined stories on the second day differed greatly between conditions, with self-reported arousal on average being 38.33 points higher (*CI*_95_ = [32.69, 43.96], *t*_32_ = 13.84, *p* < 0.001) and valence 44.75 points lower (*CI*_95_ = [40.05, 49.45], *t*_32_ = 19.39, *p* < 0.001) for threat trials compared to neutral trials. A 2 × 2 repeated measures ANOVA with Condition and Day as factors revealed decreased encoding vividness of premise memories for threat trials (*η*_*p*_^2^ = 0.12, *CI*_90_ = [0.04, 0.23], *F*_1,96_ = 13.28, *p* < 0.001). This effect was driven by a difference between conditions on day two (difference = − 8.42 points, *CI*_90_ = [− 12.80, − 4.04], *t*_96_ = − 3.82, *p* < 0.001) that was absent on day one (difference = − 2.94 points, *CI*_90_ = [− 7.32, 1.43], *t*_96_ = − 1.34, *p* = 0.185), indicating that the negative stimuli used for episodic threat conditioning may have impaired participants’ ability to imagine stories involving both elements (B → C).

### Primary analyses

#### Memories of threat learning impair associative inference

**Figure 3:**
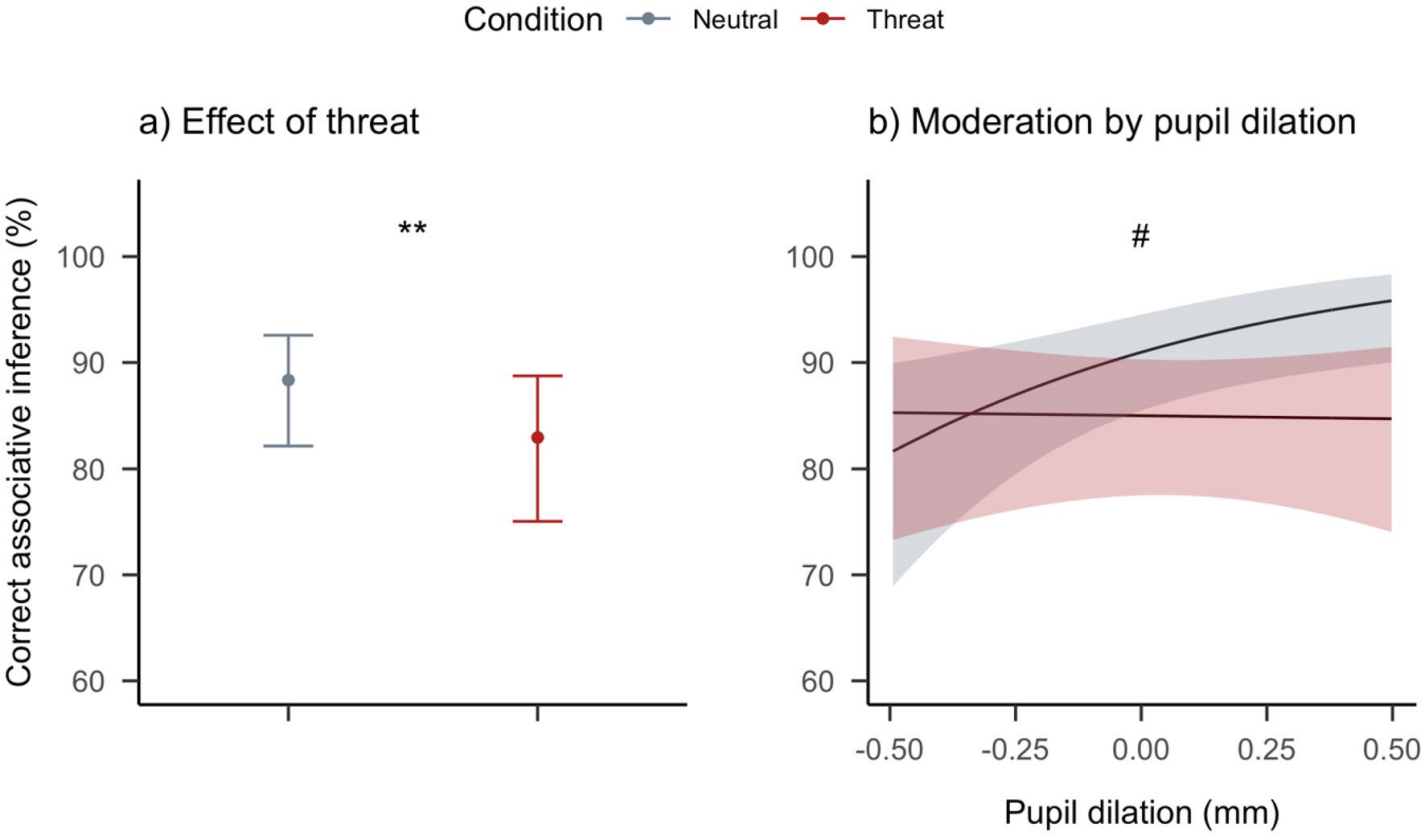
Results for both of the main hypotheses. (**a**) Threat learning significantly decreases the probability of correct associative inference. (**b**) Arousal induced by the C stimuli, here measured as a response in pupil dilation, had an enhancing effect on associative inference, but only for neutral trials. Error bars and shading represent 95% confidence intervals. Asterisks indicate statistically significant differences between conditions (*p < 0.05, and **p < 0.01), and hashtags indicate statistically significant interactions with condition (^#^*p* < 0.05).

To test the main hypothesis that threat learning memories affect subsequent associative inference we first ran a multilevel logistic regression model to estimate the effect of threat (categorical variable, neutral or threat) as the only predictor of correct or incorrect associative inference. This revealed that threat reduced the log odds ratio of correct associative inference by − 0.44 (*CI*_95_ = [− 0.78, − 0.11], *p* = 0.008), and an intercept of 2.03 (*CI*_95_ = [1.53, 2.57], *p* < 0.001). As the intercept here represents the reference condition, the log odds ratio for correct associative inference was 2.03 in the neutral condition and 2.03–0.44 = 1.59 in the threat condition (see Fig. 3a). In terms of probability, this corresponds to a 5.13% decrease in accuracy (*CI*_95_ = [1.18, 10.66]) for threat trials relative to 88.39% correct for neutral trials. This first analysis provides convincing evidence that memories of learned threat impair associative inference.

A multilevel regression analysis of reaction times revealed that participants were faster when making correct as compared to incorrect associative inferences (difference = − 9.37 s, *CI*_95_ = [− 11.50, − 7.24], *t* = − 8.62, *p* < 0.001). There was however no effect of condition (difference = 1.38 s, *CI*_95_ = [− 1.12, 3.88], *t* = 1.08, *p* < 0.281), nor an interaction effect of condition × correct on RTs (*β* = − 1.29 s, *CI*_95_ = [− 4.05, 1.47], *t* = − 0.914, *p* < 0.361), indicating no evidence for an accuracy-response time trade-off or avoidance of threat trials by spending less time on their retrieval. Finally, we tested whether average RTs for inference trials differed from both types of premise memory trials on the final day of the experiment by means of a 3 × 2 repeated measures ANOVA, using associative test type and condition as factors. There was a main effect of associative test type (*η*_*p*_^2^ = 0.67, *CI*_90_ = [0.60, 0.72], *F*_2,160_ = 162.51, *p* < 0.001), but not of threat (*η*_*p*_^2^ = 0.01, *CI*_90_ = [0.00, 0.05], *F*_1,160_ = 1.39, *p* = 0.240). Associative test type and threat did not interact (*η*_*p*_^2^ = 0.01, *CI*_90_ = [0.00, 0.04], *F*_2,160_ = 0.92, *p* < 0.399). Comparisons of marginal model means showed that inference trials averaged across conditions (RT = 9.13 s, *CI*_95_ = [8.26, 10.00]) were substantially slower than premise associations from both day 1 (difference = 5.25 s, *CI*_95_ = [4.35, 6.15], *t*_160_ = 13.84 , *p* < 0.001) and day 2 (difference = 6.42 s, *CI*_95_ = [5.25, 7.32], *t*_160_ = 16.93, *p* < 0.001), indicating no evidence for integrated representations of all trial elements (A → B → C).

#### Pupil dilation does not moderate the effect of threat on associative inference

We then analyzed whether initial arousal responses (operationalized here as pupil dilation) upon first encounter with an aversive C item were parametrically associated with the impairment by threat learning memories on associative inference. Including these variables in the model revealed an effect of arousal (*Log OR* = 1.65, *CI*_95_ = [0.39, 2.96], *p* = 0.010), and an interaction effect of arousal and condition (*Log OR* = − 1.70, *CI*_95_ = [− 3.39, − 0.05], *p* = 0.042) on subsequent associative inference (see Fig. 3b). Contrary to our hypothesis however, the positive log odds ratio parameter of arousal indicates an *enhancing* effect on associative inference for neutral trials. The negative log odds ratio parameter, of similar size, for the interaction × condition parameter indicates that this interaction is mostly driven by the neutral trials, while no effect for threat trials exists (see Fig. 3b). Indeed, there was no evidence for a relationship with arousal and associative inference for threat trials (*Log OR* = − 0.05, *CI*_95_ = [− 1.08, 0.98], *p* = 0.929). The interpretation that threat cancels the enhancing effect of arousal was further confirmed by testing the hypothesis that the parameter estimates for the main effect of arousal and its interaction with condition sum up to zero (χ^2^_(1)_ = 0.01, *p* = 0.929).

### Secondary analyses

#### High vividness at encoding for both premise memories enhances associative inference for neutral trials, but not threat trials

We tested the potentially confounding role of vividness at encoding that may interact with threat learning in its subsequent effect on associative inference. The vividness scores for both days were added to the initial model of hypothesis 1, allowing for every possible interaction with condition (vividness day 1 × vividness day 2 × condition). The model showed no main effects or interactions with condition for vividness on either day one or two. However, there was a significant three-way interaction between all predictors (*Log OR* = − 0.50, *CI*_95_ = [− 0.86, − 0.18], *p* = 0.003), indicating a difference between conditions in how associative inference is affected by vivid encoding when it is high on both days (see Table 1 for all parameter estimates). To better interpret this result, we split the dataset by condition and ran the model again on both neutral and threat trials separately, with only vividness scores for both days as predictor variables. This revealed a positive interaction effect between vividness on day 1 and day 2 for neutral trials (*Log OR* = 0.43, *CI*_95_ = [0.18, 0.72], *p* = 0.002) which was absent for threat trials (*Log OR* = − 0.08, *CI*_95_ = [− 0.28, 0.11], *p* = 0.413). These findings suggest that when all elements are neutral, associative inference is enhanced if both premise associations are vividly encoded, whereas trials that involve threat do not benefit from vividness in this manner.

**Table 1:** Multilevel regression output corresponding to the moderating role of premise memory vividness.

Variable	*Log OR*	*CI*_95_	*p-*value
Intercept	2.09	[1.57, 2.67]	** < 0.001**
Threat	− 0.43	[− 0.78, − 0.08]	**0.016**
Vivid_D1	0.26	[− 0.03, 0.53]	0.066
Vivid_D2	− 0.08	[− 0.37, 0.19]	0.539
Threat × Vivid_D1	− 0.15	[− 0.50, 0.22]	0.418
Threat × Vivid_D2	0.29	[− 0.07, 0.66]	0.111
Vivid_D1 × Vivid_D2	0.44	[0.17, 0.74]	**0.002**
Threat × Vivid_D1 × Vivid_D2	− 0.50	[− 0.86, − 0.18]	**0.003**

*CI*_*95*_ 95% confidence interval around the point estimate.

Bold letters indicate significant (< 0.05) p-values.

#### Non-specific threat detection

When confronted with potential threat, every bit of information from previous experiences can be relevant to deal with the situation. Yet, detailed retrieval of threat-associated memories may not always be required to initiate an adequate response, and could even be counterproductive when retrieval of precise details comes at the expense of costly time needed to take avoiding action. In such cases it is sufficient to infer simply that there is a threat regardless of the specifics. This only requires the generalization of negative value from C-items to their indirectly associated A-items, an effect that has previously been demonstrated in humans using sensory-preconditioning paradigms^17,18,46^. Moreover, experiments have shown that activity along the long axis of the hippocampus reflects a gradient of resolutions at which one or multiple events can be retrieved^47^, suggesting that the effect of threat on associative inference could be different at the ‘gist-level’. To test this idea, we redid the first primary analysis, but this time a response was also considered accurate when a wrong C-item originating from the correct condition was selected. This, however, did not change the pattern of results. For neutral trials, the log odds ratio of selecting a neutral item was 2.62 (*CI*_95_ = [2.17, 3.16]), corresponding to 93.24%, whereas for threat trials the probability of correctly selecting a threat item was significantly lower at 89.39% (*Log OR* = − 0.49, *CI*_95_ = [− 0.89, − 0.10], *p* = 0.013). Similarly, there was again no effect of threat on reaction times (difference = − 0.24 s, *CI*_95_ = [− 3.62, 3.15], *t* = − 0.14, *p* = 0.892), nor an interaction between threat condition and inference accuracy (*β* = 0.80 s, *CI*_95_ = [− 2.79, 4.38], *t* = 0.436, *p* = 0.663),. Complementary to this analysis, we applied methods from signal detection theory to disentangle sensitivity to threat from a potential pre-existing response bias. For a threat trial, correctly choosing any threat item constitutes a hit whereas choosing a neutral item counts as a miss. Similarly, for a neutral trial, correctly choosing any neutral item constitutes a correct rejection whereas choosing a threat item counts as a false alarm. The average sensitivity across participants was *d*ʹ = 2.49 (*CI*_95_ = [2.13, 2.83]), and we found no evidence of a response bias towards threat (*c* = 0.07, *CI*_95_ = [− 0.03, 0.17], *p* = 0.145). Consistent with the latter, an analysis of inaccurate associative inferences showed that the odds of wrongly selected C-items belonging to the correct condition did not differ from chance level (2/5, or *Log OR* = − 0.41) for either neutral (*Log OR* = − 0.30, *CI*_95_ = [− 0.80, 0.17], *p* = 0.653) or threat trials (*Log OR* = − 0.54, *CI*_95_ = [− 1.00, − 0.13], *p* = 0.538). These findings suggest that errors in associative inference were not biased towards C-items of similar value through either generalization or differences in the resolution at which threat memories are recombined, and were likely due to other mnemonic processes.

#### Original memories are enhanced following novel threat learning

We tested the hypothesis that novel threat learning may strengthen associated elements of a pre-existing, reactivated memory, in line with earlier observations^13^. Neutral associations that were later linked to an aversive item were significantly better retained on the final associative recognition test (*Log OR* = 0.454, *CI*_95_ = [0.085, 0.827], *p* = 0.016).

#### No difference between threat learning and neutral associations

Finally, we investigated whether there were differences in associative memory strength between the threat learning events and neutral controls. Particularly, we expected to find an impairment of threat^23^. However, no effect was observed (*Log OR* = − 0.235, *CI*_95_ = [− 0.785, 0.315], *p* = 0.402). Note however that the log odds ratio of the intercept (4.30) is very high, corresponding to a hit-rate of 98% in the reference condition. This can be considered performance at ceiling, masking the potential effect of threat learning on associative recognition.

## Discussion

To successfully navigate a complex world, the ability to draw new connections between distinct memories that overlap in content can be essential. Here we show that when a neutral memory is later indirectly linked to threat, associative inference is impaired whereas the initial memory is retroactively strengthened. Contrary to our prediction, we found no evidence that pupil dilation during threat learning correlated with the magnitude of associative inference impairments by threat. In fact, pupil dilation in response to C-items was actually associated with an increased probability of accurate inference for neutral trials. Similarly, high encoding vividness of premise memories was found to increase the probability of accurate associative inference for neutral trials, but not threat trials. Together these results demonstrate that episodic threat learning not only hampers associative inference, but also nullifies the beneficial effects of noradrenergic arousal and memory vividness at time of encoding. We hypothesized that associative inference would either be enhanced by episodic threat learning through prioritized integration, or impaired through disrupted binding of reactivated elements and novel experience. The present findings are consistent with the latter.

Importantly, the impairment by episodic threat memories on associative inference cannot be explained by emotional alterations of other cognitive processes. First, stimuli to be associated were presented sequentially, thereby precluding the possibility that emotional stimuli would draw disproportional attention at the expense of neutral material and the associations between them. Second, by restricting our analyses to trials for which both premise memories were still remembered on the final day, we ruled out the possibility that they were never encoded or no longer retrievable. As such, the impairment by episodic threat learning on associative inference cannot be explained by alterations in premise memories, but must be due to emotional effects in recombining information that was readily accessible.

Diminished associative binding of emotional events has been explained as resulting from increased amygdalar processing of arousing information, which leads to hippocampal overload^22^. Given that the representations required for associative inferences are particularly dependent on hippocampal activity both at encoding and retrieval^3^ they may be especially sensitive to such disruptions. Here, the indirect association between a reactivated element A and a novel, emotionally arousing item C, could have been impaired in just the same way as a direct association. However, central to this hypothesis is that such an impairment would be graded by noradrenergic arousal, but we found no evidence that threat-induced pupil dilation relates to the extent of impairment. Notably, despite the commonly held notion that noradrenergic arousal subserves alterations in associative memory^20^, arousal is rarely both indexed physiologically (e.g., using pupillometry) and analyzed on a trial-by-trial basis as we did here. It is therefore likely that the effects of arousal on associative memory, and in particular our understanding thereof, have been overstated. Surprisingly, for associative inference of neutral trials we did find an enhancing effect of pupil dilation. The reason may be that variance in pupil dilation responses, thought to reflect a summation of various cognitive processes^48^, in the neutral condition reflected a combination of arousal, effort, and elaboration that is beneficial to associative memory^49^. Indeed, recent neuroscientific studies report that pupil dilation in fact does not align with mere locus coeruleus-driven noradrenergic arousal^50,51^. Since participants often reported that it was challenging to comply with the instruction to imagine stories when presented with highly aversive stimuli (also reflected in reductions in vividness), effort and elaboration were likely absent in the threat condition, nullifying the effect of arousal.

Another mechanistic explanation for impairments in emotional associative memories attributes the decreased accuracy to lacking involvement of the parahippocampal and entorhinal cortices, structures that unitize separate items in memory^52^. In this view, the present impairment of associative inference by threat results from disturbed unitization of a reactivated A item with a newly presented emotional C item. This hypothesis is supported by the finding that reactivated elements of memories recirculate back into the entorhinal cortex to be processed as input^53^, meaning that A and C could in principle be unitized like regular associations. An enhancing effect of premise memory unitization has previously been shown for other relational memory tasks^54^. Indeed, we found that when vividness at encoding, a common measure of unitization^55^, is high for both premise memories, the odds of successful associative inference are increased, but *only* for neutral trials. These findings indicate that the disturbance of unitization processes both at the level of direct associations within premise memories, as well as between reactivated and new items may underlie the impairment of associative inference by threat memories.

Although both mechanisms described above are sufficient to explain the impairment of episodic threat learning on associative inference, neither accounts for the observed enhancement of associations within initially neutral premise memories. This finding is consistent with the results of Zhu et al.^13^, who showed that enhanced associative memory following emotional tagging may be supported by integration. However, the fact that in the present study initial memory was enhanced while associative inference was impaired makes it unlikely that predictive associative chains of items (A → B → C) were stored and retrieved as single integrated representations. This is supported by the very slow reaction times for associative inference relative to associative recognition within premises, as fast judgements are considered a hallmark behavioral expression of integrated premise memories^9^. Moreover, our incidental encoding instructions may not have triggered an integration state, which is qualitatively distinct from encoding and retrieval states^56^. In sum, memory integration cannot explain neither the impairment of associative inference, nor the retroactive enhancement of initial memories by threat learning.

To separately account for the observed retroactive enhancement of initial memories, we tentatively suggest that their retrieval and novel association with threat on day two tags the synapses involved for subsequent strengthening^57^. Importantly, this observation differs from previous reports of retroactive memory enhancements^12,58^. For one thing, earlier studies timed their emotion manipulation shortly following encoding, while here memories were specifically reactivated and manipulated well after the consolidation window had closed. Moreover, in those studies, memory elements belonged to two distinct superordinate categories, one of which was later made relevant by means of threat conditioning. Here, in contrast, the elements that made up the initial memories did not belong to any distinct categories, and were newly associated to trial-unique USs. The link through which retroactive enhancement occurred was therefore strictly episodic rather than semantic, suggesting a new avenue by which retroactive memory enhancement can be achieved, well beyond the original window of consolidation. It must however be noted that the associative recognition test of initial memories occurred after the associative inference test, which was the primary interest of this study. An alternative explanation then is that the emotional enhancement only happened a few minutes before testing when the initial memories were consciously recombined with memories of threat, and is therefore independent of their reactivation in the presence of threat the day before. Given the manifold implications of selective retroactive memory enhancement by emotion^58^, more work is required to assess the reliability of this finding and investigate its underlying mechanisms.

It is important to note that although the PRE-memory paradigm was inspired by sensory preconditioning studies, we can currently only speculate on whether value generalization has occurred, and how this relates to declarative memory recombination, since we have not measured arousal to A stimuli on day 3. Yet, analyses of reaction times and accuracy at the gist level provide some information that is relevant to these outstanding questions. An important feature of physiological responses to learned predictors of threat is that they are expressions of an automatic associative learning system that quickly recruits defensive mechanisms in the face of danger^59^. From this perspective, it could be predicted that defensive responses would generalize to A, on their turn promoting inference of threat. If value generalization were to have occurred in the present experiment, we would have expected faster response times, both at the specific and general threat level, and higher gist-level accuracy for threat. Our secondary analyses, however, provide no evidence for such an effect, suggesting that value generalization did not occur. Given the impairing effect of threat, it seems also unlikely that value generalization still somehow benefitted associative inference.

These observations contradict earlier human sensory preconditioning studies that demonstrated generalization of threat even when the two cues presented during pre-conditioning are conceptually unrelated^15^, and when multiple A-B associations need to be learned^60^, as was also the case here. However, the PRE-memory paradigm differs from earlier work as it requires first a mapping of many unique A stimuli to equally many B stimuli, which are subsequently linked to unique reinforcers (C stimuli). It is therefore possible that the large amount of unrelated stimuli, all part of unique episodes, created too large a cognitive demand for generalization to occur through a ‘simple’, model-free associative learning process^61^, and participants instead relied purely on hippocampus dependent cognitive maps of A-B-C associations. For a definite conclusion on whether generalization occurs when cognitive demand is high, and whether this affects associative inference, it will be necessary to run the litmus test of affective generalization using the present design, but incorporating a different test phase where A → C pairs are presented, thus allowing the assessment of conditioned responses to A.

In conclusion, we here reveal that memories of threat learning hamper associative inference. Even though at first sight it may be particularly adaptive to connect past neutral memories with related threat learning events, humans may be protected against such episodic linking of threat by a mechanism that compartmentalizes, rather than integrates, negative experiences, causing impairments in associative inference. This however raises the intriguing question of whether this mechanism is to some degree defective in clinical populations. Our memories form the foundation for our predictions and simulations of the future^2^, and if these are based on a memory system that does not protect itself from needlessly integrating episodic threat memories into its web of connections, the result could be maladaptive rumination and anxiety. The present study shows that a healthy memory may actively prevent the unnecessary linkage of reactivated elements to threat, both specifically or as a general category, thus potentially safeguarding against such symptoms. Note however that this form of episodic threat linkage is fundamentally different from affective value generalization as typically discussed in threat conditioning experiments. It concerns the potential conscious reliving of negative events, instead of a psychophysiological experience of heightened arousal, when facing or remembering an indirect predictor of threat based on a cognitive map of related stimuli and events. Whether value generalization and associative inference are related or may even interact to optimally prepare for an anticipated threat remains an intriguing question to be investigated in future studies.
